# Pressure sores–a constant problem for plegic patients and a permanent challenge for plastic surgery

**Published:** 2010-05-25

**Authors:** C Giuglea, S Marinescu, IP Florescu, C Jecan

**Affiliations:** Plastic and Reconstructive Surgery Clinic, ‘Bagdasar–Arseni’ Emergency Hospital, BucharestRomania

**Keywords:** pressure sores, plegic patients, surgical treatment, neuromuscular recuperation treatment

## Abstract

Pressure sores–a constant problem for plegic patients and a permanent challenge for plastic surgery

Pressure sores can be defined as lesions caused by unrelieved pressure resulting in damage of the underlying tissue. They represent a common problem in the pathology of plegic patients and, plastic surgery has a significant role in their treatment. Pressure sores occur over bony prominences and so, they are most commonly seen at the sacrum and trochanters in paralyzed patients and at ischium for the patients who sit in a wheelchair for a long time.

For these patients, surgical treatment is very important because on one hand, it stops the loss of nutrients and proteins at the site of the pressure sore, and on the other hand, it permits the initiation of neuromuscular recuperation treatment much faster.

## Introduction

In the beginning of this paper, we would like to present a few general data about pressure sores: they can be defined as lesions caused by unrelieved pressure resulting in damage of the underlying tissue; the prevalence of pressure sores in acute care units is about 10 %, and in chronic care units this prevalence can be between 3.5 and 50%.  More than 60% of the pressure sores occur in elderly patients of over 70 years old and the majority of patients with pressure sores are either paralyzed, elderly or hospitalized.

The incidence in plegic patients is of about 21% for paraplegics and 23% for quadriplegics. [1] They represent the cause of death for 7–8% of paraplegic patients.

Pressure sores occur over bony prominences and so, they are most commonly seen at the sacrum (36 to 60%), ischium (6%), trochanters (6%), heel (30%).[2,3]

They can be classified as it follows:

- Stage one: nonblanchable erythema of intact skin
- Stage two: partial thickness skin loss involving epidermis, dermis, or both. The ulcer is superficial and it clinically presents as an abrasion, blister or shallow crater
- Stage three: full thickness skin loss involving damage or necrosis of subcutaneous tissue that may extend down to but not through underlying fascia; it clinically looks like a deep crater with or without undermining of adjacent tissue
- Stage four: full thickness skin loss with extensive destruction, tissue necrosis or damage of muscle, bone, or supporting structures.[4]

The formation of a pressure sore is a result of an increased tissue tolerance as mediated by certain intrinsic and extrinsic factors, when a pressure is applied on a defined duration and intensity.[5]

**Figure 1:**
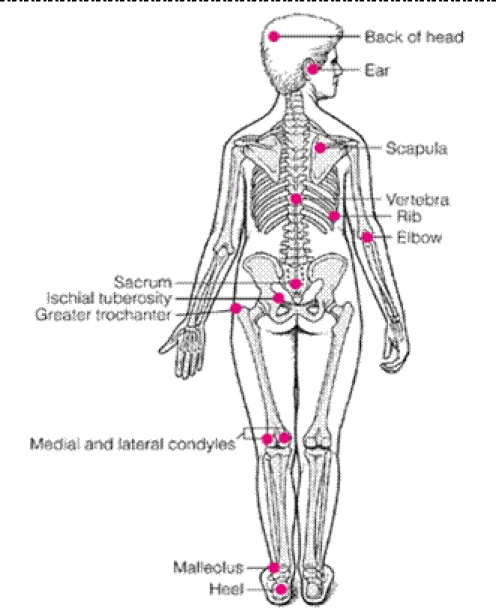
Skeleton presentation–Common sites of pressure ulcers. The most common sites are the sacrum, greater trochanters, ischial tuberosities, medical and lateral femoral condyles, malleoli and heels. Other sites include the elbows, scapulae, vertebrae, ribs, ears and back of the head.
  The best way to treat a pressure sore is to prevent it! Reducing the pressure can prevent many of the pressure sores; it can be obtained by changing the position of the patient at every 2 hours. Using special air–fluidized beds that can reduce the pressure bellow 25 mm Hg with equal distribution or special pillows for wheelchairs, avoiding moisture by changing the bed sheets often, can also help in preventing their formation.[8]
- **Surgical treatment** has an important role in solving this problem but it is not always the first option.
  The right way to treat a pressure sore is to make a good plan, meaning a good general condition of the patient in order to obtain a good local healing combined with the best surgical option for the affected area. The patient should be prepared for pressure sore surgery much as for any other large surgical procedure: adequate nutrition, absence of concomitant infection, optimization of any other medical conditions.[6]
  The principles for pressure sore surgery were first described by Conway and Griffith in 1956:
  - complete excision of the sore, surrounding scar and underlying bursa is first performed
  - the underlying bone is removed until healthy bleeding bone remains
  - the resultant death space is filled with large pedicled flaps, either fasciocutaneous or myocutaneous
  - it is better to have a large flap design in order to avoid sutures in the pressure areas
  - also avoid violation of adjacent flap territories, which may be needed for future use.[9]
  The common procedures employed to close pressure sores located in sacral, ischial and trochanteric regions will be presented as being the most frequent situations plastic surgery has to deal with. For some areas and in different clinical situations we can use myocutaneous flaps which are well–vascularized and have enough tissue to fill large death spaces, or fasciocutaneous flaps used especially for sacral sores.[10]
  We will present the most frequent situations in which plastic surgery has a significant role.

Primary factors are:

- **Pressure**, more than 32 mm Hg leads to ischemia; even though in dorsal decubitus  the pressure is more than 32 mm Hg, changing the position prevents pressure sore formation; muscle is more sensitive to ischemia, so muscle necrosis can occur much faster than skin necrosis. [6] Tissue tolerance: extrinsic factors like increased moisture, friction and shear and intrinsic factors like poor nutrition (patients with pressure sores need a hypercaloric diet 1.5–3.5g/Kg/day proteins and 25–35  nonproteic kcal/Kg/day, vitamin C, A, and other minerals involved in wound healing), age (older people have a low vascularity of the skin), anesthesia (for patients with spine lesions), wound infection (all ulcers are infected!), immobilization leads to contractures that induce unequal distribution of pressure, low arteriolar pressure, systemic diseases like diabetes, neurologic disorders, smoking.[7]
  The common sites where pressure sores can appear are as it is shown in the next figure[Figure 1]:

**Surgical treatment of a sacral pressure sore** requires adequate soft–tissue debridement and excision of any bony irregularities.

Fasciocutaneous flaps used in this area include random rotation/advancement flaps and transverse lumbosacral back flaps,[11] the use of these flaps has the advantage of avoiding loss of function associated with muscle flaps, and also avoids placing the sutures in pressure area, being the first choice in nonparalyzed patients. 

Myocutaneous flaps are represented by the unilateral or bilateral gluteus maximus flap advanced medially in a V–Y fashion; the gluteus muscle can be also used as an island flap[12] in order to preserve muscle function;[13] these flaps are more commonly used in paralyzed patients or in cases with large, deep defects.

In the treatment of **ischial pressure sores** careful consideration should be given to the ischial ramus. Both fasciocutaneous and muscle flaps were described for this area. For paralyzed population, the V–Y advancement of the hamstring muscles as myocutaneous flap has proven highly effective.[14]

Other muscle flaps available for this region include the inferior gluteus maximus, gracilis, and rectus abdominis.[15, 16, 17, 18]. For ambulatory patients, the posterior gluteal thigh flap is the treatment of choice, this flap is an axial fasciocutaneous flap based on a subfascial descending branch of the inferior gluteal artery.[19]

Tensor fascia lata flap can also be useful in ischial sores and it can be a sensate flap based on the lateral femoral cutaneous nerve.[20] 

Direct closure of **trochanteric sores** is not usually possible because of the greater tension of lateral hip tissue. The flap of choice for this area is tensor fascia lata, its classic use being posterior transposition, but it can be used as a V–Y advancement flap or in combination with gluteus medius [20, 21]. Other techniques described for this area include a distally based gluteus maximus flap, or a vastus lateralis myocutaneous flap and gluteal thigh flap [22].

**Postoperative care** consists in:

- attention to pressure relief and wound care
- pharmacologically induced constipation in those patients who do not have a colostomy[23]
- suction drains are maintained until they produce less than 10 to 15 cc per day

Patients should remain in the hospital for at least one week and may then be discharged if appropriate pressure relief and continued wound care is available. In the first few weeks after pressure sore surgery problems with hematomas, recurrent infection and wound dehiscence can be seen.

Many of these problems can be avoided by strict attention to technical details and by respecting the general principles of flap design and elevation. 

## Material and methods

### Used materials

In the last ten years, we have performed surgical treatment for pressure sores of the patients admitted in our hospital. Their number was 200. 57% out of them were paraplegic, 38% were quadriplegic, and 5% were ambulatory.

The most frequent sites we operated on were:

- sacral sores– 48%
- ischial sores– 32%
- trochanteric sores– 20%

### Work methodology

After the admission of each patient, we analyzed the blood in order to correct any disturbances and obtain values close to normal. When patients had a good metabolic condition and with a good control of the infection we performed the operation considering the site of the pressure sore, the status of the patient and the local resources.

Therefore, after the patient was well prepared for the operation, having a blood reserve in order to supply the blood loss during the sore excision and flap elevation, we performed the operation under general anesthesia or no anesthesia for the patients with spinal cord lesions.

Considering the condition of the patient (ambulatory or plegic) we chose the coverage option in order to obtain the fastest recovery and the most durable result.

For sacral sores, we used either fasciocutaneous flaps or myocutaneous flaps.

For ischial sores, we preferred myocutaneous flaps, having to deal with very deep and infected sores; we considered this the best option for the patient.

For trochanteric sores, we thought the best option remained the tensor fascia lata myocutaneous flap.

## Results and discussions

Using a specific method of coverage for each defect, allowed us to obtain good results, with minimal complications and durable results. We appreciate that plastic surgery has a significant role in pressure sores treatment, our results being most satisfactory not only for the patient but also for the surgeon and kinetotherapist.

Considering the quick recovery of the patient who needs rehabilitation, we think that surgical treatment proved to be mandatory. With a good cooperation of the patient and in a good metabolic condition, surgical treatment can put an end to the patient's problems and start a strong rehabilitation program.

**Figure 2:**
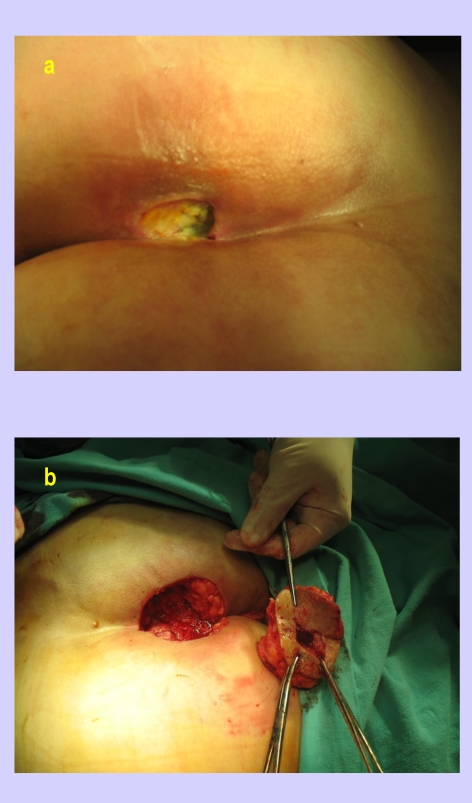
Sacral sore–preoperative aspect and excision

**Figure 3:**
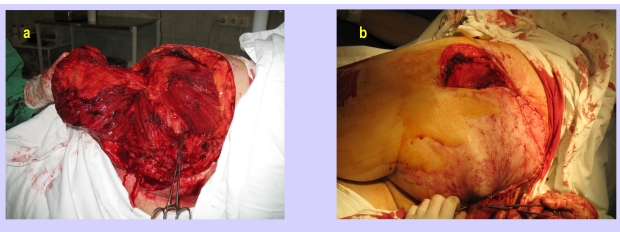
Sacral sore–Intraoperative aspects–miocutaneous gluteal flap

**Figure 4:**
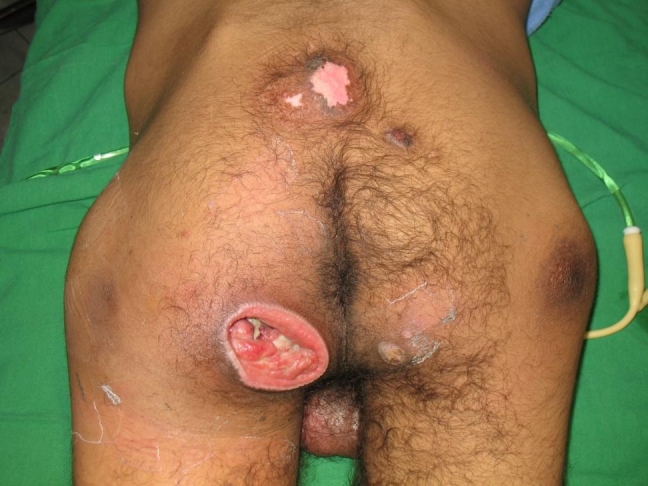
Ischial sore–preoperative aspect

**Figure 5:**
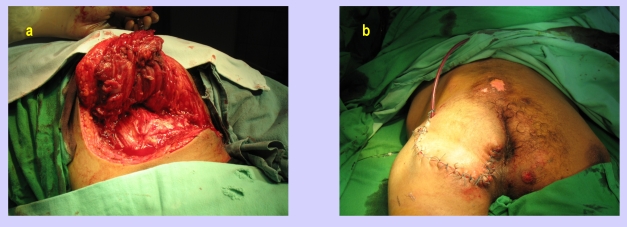
Ischial sore–intraoperative aspect

**Figure 6:**
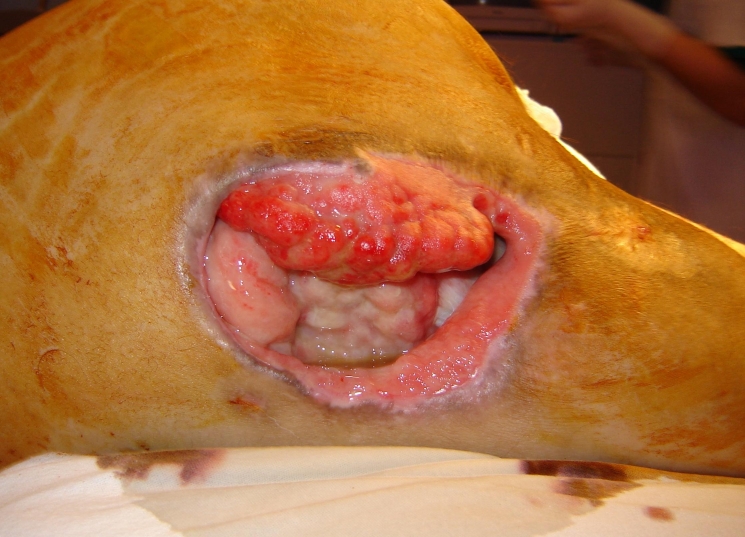
Trochanteric sore–preoperative aspect

**Figure 7:**
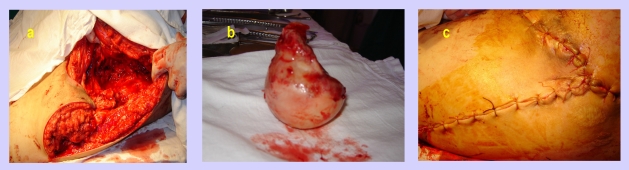
Trochanteric sore–intraoperative aspects

In the next figures, we will present a few cases and their surgical treatment:[Figure 2, Figure 3,Figure 4,Figure 5,Figure 6,Figure 7

## Conclusions

Pressure sore surgery remains a challenge for plastic surgeons in their attempt to solve these problems so frequent in plegic patients. With a good knowledge of surgical techniques and particularities of each region to be treated, we can obtain very good results. 

Pressure sores can be a pathology that slows down the patients who need rehabilitation procedures in their program of recovery. That is why a quick solving of their problem can be very helpful. It is best to choose the simplest way to solve a case in order to minimize complications and obtain a satisfactory result. 
